# Plastic surgeons as COVID doctors during second wave of COVID-19 in India

**DOI:** 10.1016/j.ijscr.2021.106130

**Published:** 2021-06-24

**Authors:** Naveen Kumar

**Affiliations:** Department of Plastic Surgery, Lady Hardinge Medical College and Associated Hospital, New Delhi 110001, India

**Keywords:** COVID-19, Plastic surgeons, Second wave, Doctors, Treatment

Sir,

Today we are witnessing the second wave of The COVID-19 pandemic in India that has completely disrupted the lives of large section of the society. April month had witnessed a steep peak in the number of daily active cases and unfortunate deaths across the country. And even today i.e. at the end of first week of May while writing this letter, we are seeing about 4 Lakh of active cases with around 4000 of deaths coming within 24 hours. Unfortunately, this has devastated our normal surgical and routine clinical practice. We are no longer considering ourselves as a plastic surgeon but a COVID doctor, since our role at this moment of time is to save the lives of anyone getting trapped in this tsunami of COVID-19. Many of plastic surgery residents and consultants are being regularly deployed by the hospitals as front line workers against such emergency. Apart from working in institutes and hospitals, many of us are experiencing that a large number of people are seeking advice and treatment through phone calls and social media for the signs and symptoms surprisingly not related to our specialty but specific to the COVID-19 disease. Seeing such scenario during this second wave, we had conducted an online survey (using Google forms) across the country through the social media network (WhatsApp, Facebook messenger) and direct emails in order to gather the experience of the plastic surgeons in India using set of questions. The survey has gathered the responses from 30 (61.2%) plastic surgery residents and 19 (38.7%) consultants.

When asked how much prepared were they in the second wave as compared to the first one? 40/49 respondents with 27 (90%) residents versus 13 (68.4%) consultants believed themselves as more prepared than earlier. 35 of the total participants (100% residents versus 84.2% consultants) had agreed that they had undergone some sort of training program for the management of COVID-19 over the past one year. When asked, majority of the participants (97% residents versus 95% consultants) had agreed to have knowledge of updated treatment protocol as per the guidelines led by the government agencies and ICMR [1] during the second wave (47/49). Interestingly, when asked how often patients had contacted either via phone calls or social media in one day during the month of April for seeking the advice for the COVID-19? 20 (40% residents versus 42% consultants) of the participants had replied with maximum 5 times, 12 (30% residents versus 16% consultants) had replied between 5–10 times, 8 (13% residents versus 21% consultants) had said 10–15 times, and 9 (17% residents versus 21% consultants) had said more than 15 times a day.

Majority (90% residents and 95% consultants) had agreed that they had counseled and treated more patients with mild symptoms in home isolation during the month of April 2021 compared to any of the previous months since March 2020 (45/49). This data has clearly indicated the burden of the disease that the second wave has brought to the doctors irrespective of their specialty. 10 (13% residents versus 31% consultants) of the total participants had believed that the various mutant of the virus were responsible for the second wave whereas 30 (67% residents versus 53% consultants) of the respondents had agreed that the apathy of people towards COVID-19 appropriate behavior and large scale gatherings during elections and religious events were the main reasons behind the outbreak while 5 (7% residents versus 16% consultants) of the total respondents believed that both these factors were equally responsible. Majority (90% residents and 95% consultants) of the respondents were in the agreement that the imposition of strict lockdown and large scale vaccination program were essential to limit the spread of the second wave outbreak (45/49). 80% of residents had informed that they were deployed in various dedicated Covid wards, Covid ICU and Covid emergency whereas 40% of consultants had agreed to be redeployed in dedicated Covid ward of the same hospital and remaining 60% of consultants had informed that they remained in their previous wards with Covid patients. 98% of residents and 70% of consultants had informed that their working hours had increased to two to three times during the redeployment for Covid duties in comparison to their previous routine work during April 2021. This letter has emphasized upon the growing role of the doctors from other specialty like plastic surgery to combat the spreading second wave of this emergency. During this period of crisis, our priority as a plastic surgeon must be set up in order to contribute to the management of this pandemic by withholding the unnecessary elective surgical procedures and treating the patients with best of our updated knowledge.

## Consent

Informed consent was obtained.

## Consent for publication

Consent to publish has been obtained.

## Ethical approval

The study is in accordance with the Ethical Standards of the Responsible Committee on Human Experimentation (institutional or regional) and with the Helsinki Declaration of 1975, as revised in 2000.

## Funding

None.

## Guarantor

Dr Naveen Kumar

## Research registration number

Not applicable.

## CRediT authorship contribution statement

Dr Naveen Kumar - Study concept or design, Data collection, Data analysis or interpretation, Writing the paper.

## Declaration of competing interest

The authors declare that they have no competing interests.
